# High spatial and spectral resolution dataset of hyperspectral look-up tables for 3.5 million traits and structural combinations of Central European temperate broadleaf forests

**DOI:** 10.1016/j.dib.2024.111105

**Published:** 2024-11-03

**Authors:** Tomáš Hanousek, Terézia Slanináková, Tomáš Rebok, Růžena Janoutová

**Affiliations:** a Global Change Research Institute of the Czech Academy of Sciences, Bělidla 986/4a, 603 00 Brno, Czech Republic; b Department of Geography, Faculty of Science, Masaryk University, Kotlářská 2, 611 37 Brno, Czech Republic; c Institute of Computer Science, Masaryk University, Šumavská 416/15, 602 00 Brno, Czech Republic

**Keywords:** LUT, Radiative transfer model, DART, Machine learning model, Synthetic spectral data, Leaf traits, Hyperspectral data

## Abstract

Accurate retrieval of forest functional traits from remote sensing data is critical for monitoring forest health and productivity. To achieve sufficient accuracy using inverse methods it is essential to have representative database of simulated or measured spectral properties together with corresponding forest traits. However, existing datasets are often limited in scope, covering specific sites and times with simplified structures. This limitation hinders the development of generalizable machine learning models for trait prediction. To address this issue, we present a comprehensive high-resolution dataset of hyperspectral Look-Up Tables (LUT) designed for Central European temperate broadleaf forests.

The dataset includes 3.5 million unique combinations of leaf biochemical and canopy structural characteristics of forest scenes together with a variety of sun geometry. The spectral data cover wavelengths from 450 nm to 2300 nm, with a resolution of 2 nm. The dataset is organised into two files: one capturing the average reflectance of all scene pixels and another focusing solely on sunlit leaf pixels. LUT were generated using the Discrete Anisotropic Radiative Transfer model version 5.10.0. Virtual forest scenes were based on 3D tree representations derived from Terrestrial Laser Scanning of European beech trees, adjusted to various leaf area index values and structural configurations to simulate natural forest variability. The reflectance data were processed using MATLAB and Python scripts, resulting in hyperspectral cubes that were processed to generate the LUT.

The dataset can be used to train machine learning models, such as Random Forest and Support Vector Machines, for predicting forest functional traits and assisting in the calibration of remote sensing algorithms. The biggest advantage of the dataset is high spectral and spatial resolution, together with the high number of different trait combinations, which allows for adaptability to different times, locations, and hyper- and multispectral sensors, and can support up-coming hyperspectral satellite missions. ESA Copernicus Hyperspectral Imaging Mission for the Environment (CHIME) and NASA Surface Biology and Geology (SBG) future satellite missions can utilise this dataset to develop their product processors for monitoring forest traits.

Specifications Table

**Table:** 

Subject	Agricultural Sciences/Forestry Data Science/Applied Machine Learning Environmental Science/Global and Planetary Change
Specific subject area	The dataset focuses on forest modelling, exploring various states of Central European temperate broadleaf forests, and utilising radiative transfer models.
Type of data	The dataset includes two databases in .CSV format, each containing 3.5 million hyperspectral signature variations with corresponding leaf traits, structural characteristics, and sun geometry combinations. One database provides the raw format, representing the average value of the whole scene, and the second database represents the average of masked sunlit pixels.
Data collection	Look-Up Tables (LUT) were generated using DART (Discrete Anisotropic Radiative Transfer) version 5.10.0. Four different 3D tree representations with various Leaf Area Index (LAI) values of beech trees were used to set up virtual scenes. Different Sun geometry, canopy LAI, Canopy Cover, and leaf traits were set to these scenes. The reflectance of these scenes was simulated from 450 nm to 2 300 nm in 2-nm steps. A total of 3,456,000 unique combinations were generated. Images from each scene were merged into a hyperspectral cube in MATLAB r2020b and then processed in Python 3.6. Two different LUT were generated: 1) with average value of all pixels and 2) with average value of only sunlit leaf pixels.
Data source location	Institutions: Institute of Computer Science, Masaryk University; Global Change Research Institute of the Czech Academy of Sciences City: Brno Country: Czech Republic
Data accessibility	Repository name: National Repository Data identification number: 10.48700/datst.bcnpf-47q73 Direct URL to data: https://data.narodni-repozitar.cz/general/datasets/4y0sy-qh735

## 1 Value of the Data

• Look-Up Tables (LUT) are considered important training datasets for machine learning models to predict leaf traits.
• To date, only a limited number of LUT datasets have been developed for forest sites, particularly for Central European temperate broadleaf forests. Most of them are limited to a specific forest site, therefore containing only a limited combination of forest traits and specific sun geometry often using simplified forest structure [1].
• LUT presented in this study are based on virtual scenes simulated in the Discrete Anisotropic Radiative Transfer (DART) [[2], [3], [4]] model with high spatial (0.2 m) and spectral (2 nm) resolution and in high structural detail (individual leaves) .
• The large number of combinations (3.5 million) of leaf biochemical and canopy structural characteristics of forest scenes, together with a variety of sun geometry, makes the LUT general for usage at different times and locations across Europe and beyond.
• DART simulated images were processed in two ways, which provided two different LUT suitable for both satellite and airborne or drone data assessment. The high spectral resolution (2 nm) makes the LUT suitable to resample to any hyperspectral or multispectral optical sensor. Which is highly valuable for the up-coming hyperspectral satellite missions.

## 2 Background

The motivation for generating LUT was the need to sufficiently train machine learning approaches to retrieve forest functional traits, ensuring that the algorithm can work effectively on any chosen area and time in the vegetation season. While the optimal amount of training data for machine learning algorithms is not clearly established, these methods generally benefit from large heterogeneous data volumes. Similar approach of generating LUT to obtain training data has been utilized for the Eucalyptus site in Australia [5]. Nevertheless, these LUT were optimized for one specific site, time, and sensor, which makes them not well suitable for implementation to other sites or even time. To generate sufficiently general LUT for every forest type or even specific forest species can be a time- and resource-intensive task. Therefore, Central European temperate broadleaf forests, the dominant forest type in the Carpathian region [6], were focused on. To simulate various states of forests in different moments in time, leaf traits based on [7] were selected. In addition, the LUT cover various structural and scene parameters as well. The dataset provides a baseline for developing robust, reliable, and fully operational retrievals of high-quality forest functional traits from future hyperspectral satellite missions.

## 3 Data Description

The dataset consists of two files. The file “LUT_dataset.zip” contains the whole dataset, while the file “LUT_example_subset.zip” contains example subset of the dataset. In “LUT_dataset.zip” file, there are two folders. The “look_up_tables” folder contains two LUT files “LUT_all_pixels.csv” and “LUT_sunlit_pixels.csv”, comprising 3,456,000 unique simulated broadleaf forest canopy reflectance signatures. The first file contains average reflectance values of the whole scene, while the second file includes average values of only sunlit leaf pixels. “LUT_parameters.csv” file lists all combinations of variable DART input parameters used for generating the LUT. “LUT_variables_abbreviations.csv” file defines all abbreviations used in all other files.

The “codes” folder contains all the codes for processing the simulated images and generating LUT. The code “merge_images.m” converts the simulated images for individual wavelengths into one hyperspectral cubes in BSQ format for each simulation. The function “readilwis.m” facilitates this process by enabling the code to read DART .mp# image files. The code “LUT_processing.py” post-processes hyperspectral cubes (Section 4.4) and generates all files in the folder “look_up_tables” except “LUT_variables_abbreviations.csv” (Table 1).

**Table 1:** Explanation of leaf traits, structural and scene parameters abbreviations used in the “LUT_parameters.csv” file containing combinations of variable DART input parameters for the LUT generation (also in the file “LUT_variables_abbreviations.csv”).

Variable abbreviation	Variable full name
LAI	Leaf area index
CC	Canopy cover
SAA	Sun azimuth angle
SZA	Sun zenith angle
Cab	Leaf chlorophyll content
Car	Leaf carotenoid content
Cw	Leaf water content
Cm	Leaf dry matter content
N	Structural parameter

**Fig. 1:**
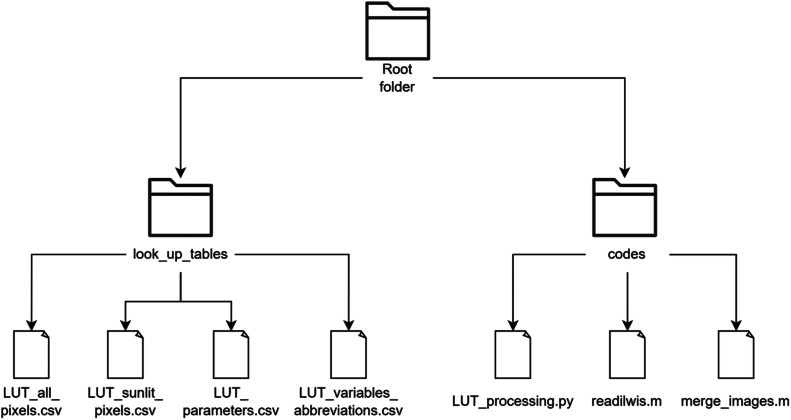
Structure of the LUT_dataset.zip data repository.

The file “LUT_example_subset.zip” has same structure as file “LUT_dataset.zip” but contains only sample of the whole dataset. Sample files include 10,000 randomly selected rows from original LUT files (“LUT_all_pixels.csv” and “LUT_sunlit_pixels.csv”) and from “LUT_parameters.csv”. Files in the folder “look_up_tables” have the prefix “subset_” for each subset file and folder "codes" contains the same files as in the whole dataset file (Fig. 1).

## 4 Experimental Design, Materials and Methods

**Fig. 2:**
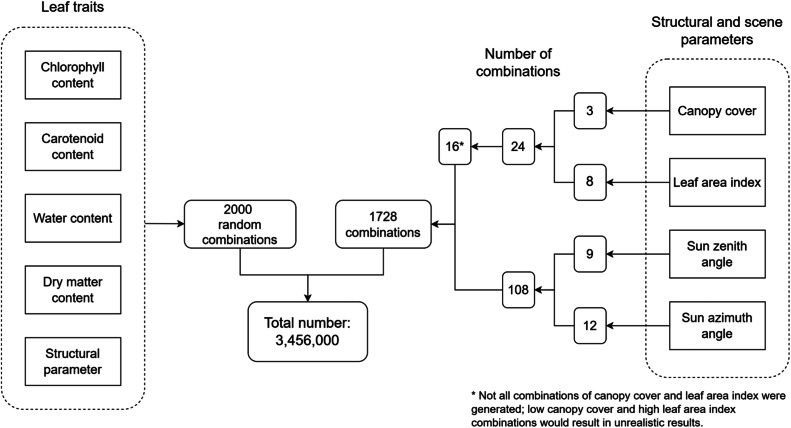
Schematic representation of the combinations of leaf traits and structural and scene parameters. The lower number of combinations from canopy cover and leaf area index are described in Section 4.2 and Table 2.

This article presents a dataset of LUT for Central European temperate broadleaf forests. The dataset comprises 3,456,000 combinations of leaf biochemical and canopy structural characteristics of forest scenes together with a variety of sun geometry (Fig. 2), which were simulated using DART [[2], [3], [4]] and representing various states of the forest in different moments in time.

The generation of LUT is based on sufficiently precise structural parametrization of a canopy (Section 4.1), correct virtual scene setting (Section 4.2), and a comprehensive dataset of traits obtained through precise in-situ measurements [7] (Section 4.3). The processing of DART simulated outputs is described in Section 4.4.

### 4.1 Tree reconstruction from Terrestrial Laser Scanning

The method proposed by [8,9] involves (i) acquiring Terrestrial Laser Scanning (TLS) data for selected tree species, (ii) segmentation of a TLS tree point cloud separating wooden parts from foliage, (iii) reconstruction of wooden parts (stem and branches) from TLS data, and (iv) biologically genuine distribution of foliage within the tree crown. Four different European beech *(Fagus sylvatica)* trees were scanned with a Riegl VZ-400 terrestrial laser scanner (Riegl LMS GmbH, Horn, Austria) at the Těšínské Beskydy site in the Czech Republic (49°35′42″ N, 18°47′31″ E, elevation 500–600 m a.s.l., [9]). Point clouds from individual scans were aligned, co-registered, and processed in RiScan Pro (RIEGL Laser Measurement Systems, Horn, Austria) .

The distribution of 3D foliage elements is derived by the tree LAI value [8]. We used a range from 4.83 to 16 to achieve defined canopy LAI values (Table 2) and therefore generated 16 unique variations of each tree, resulting in 64 individual trees in total. Trees were not converted to turbid cells but used as 3D objects. Tree LAI values higher than 16 were not used, as the point cloud density was not sufficient for the foliage distribution algorithm to be able to allocate a required number of leaves. In [10] the authors measured the highest LAI for European beech trees as 14.6. They suggested that their method overestimated LAI due to the fully closed canopies and high foliage density, leading to overlapping leaves that inflated the measured values, indicating that a value above 16 is highly unlikely to occur.

**Table 2:** LAI values for individual trees based on overall canopy LAI.

Canopy cover	60 %	75 %	90 %
Canopy LAI	Tree LAI	Tree LAI	Tree LAI
3	9.88	7.1	4.83
4	13.17	9.6	6.4
5	16	12	8
6	–	14.15	9.6
7	–	16	12
8	–	–	12.9
9	–	–	14.5
10	–	–	16

### 4.2 Virtual 3D forest scene setting and DART parameterization

To accurately simulate various states of the forest in different moments in time, it is necessary to configure correctly factors such as canopy cover (CC), canopy LAI, sun zenith angle, and sun azimuth angle. All mentioned parameters are based on values commonly present in real forest or remote sensing images [11], their ranges and steps used for generating LUT is shown in Table 3.

**Table 3:** The ranges of the structural and scene parameters and the steps in which the combinations used to generate the LUT were assembled.

Variables	Abbreviation	Start range	End range	Step	Combinations
Canopy cover [%]	CC	60	90	15	3
Leaf area index	LAI	3	10	1	8
Sun zenith angle [°]	sza	25	65	5	9
Sun azimuth angle [°]	saa	0	360	30	12

**Fig. 3:**
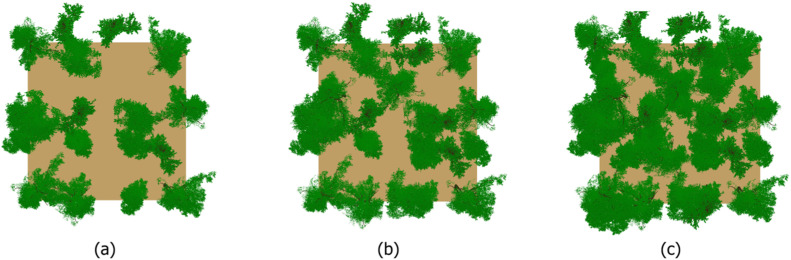
Three base scenes with different values of CC, (a) CC 60 %, (b) CC 75 %, (c) CC 90 %.

The virtual forest scenes are 30 m × 30 m large, with a cell size of 0.2 m. The dimensions of the scene were set taking into account the ability to position a sufficient number of trees, i.e. at least 18. Three base scenes were configured following the values of the CC parameter (Fig. 3). The CC 90 % (36 trees) scene was populated with trees positioned randomly, while the CC 75 % (25 trees) and CC 60 % (18 trees) scenes were set by removing trees from CC 90 % to achieve the specified CC parameter value. Assigning individual trees to a given CC value is described in Section 4.1 (Table 2). In total, 16 scenes from combinations of CC and LAI representing natural structural variability in forests were generated (Fig. 2). Repetitive scene option in DART [12] were used to enhance the authenticity of the simulation.

**Fig. 4:**
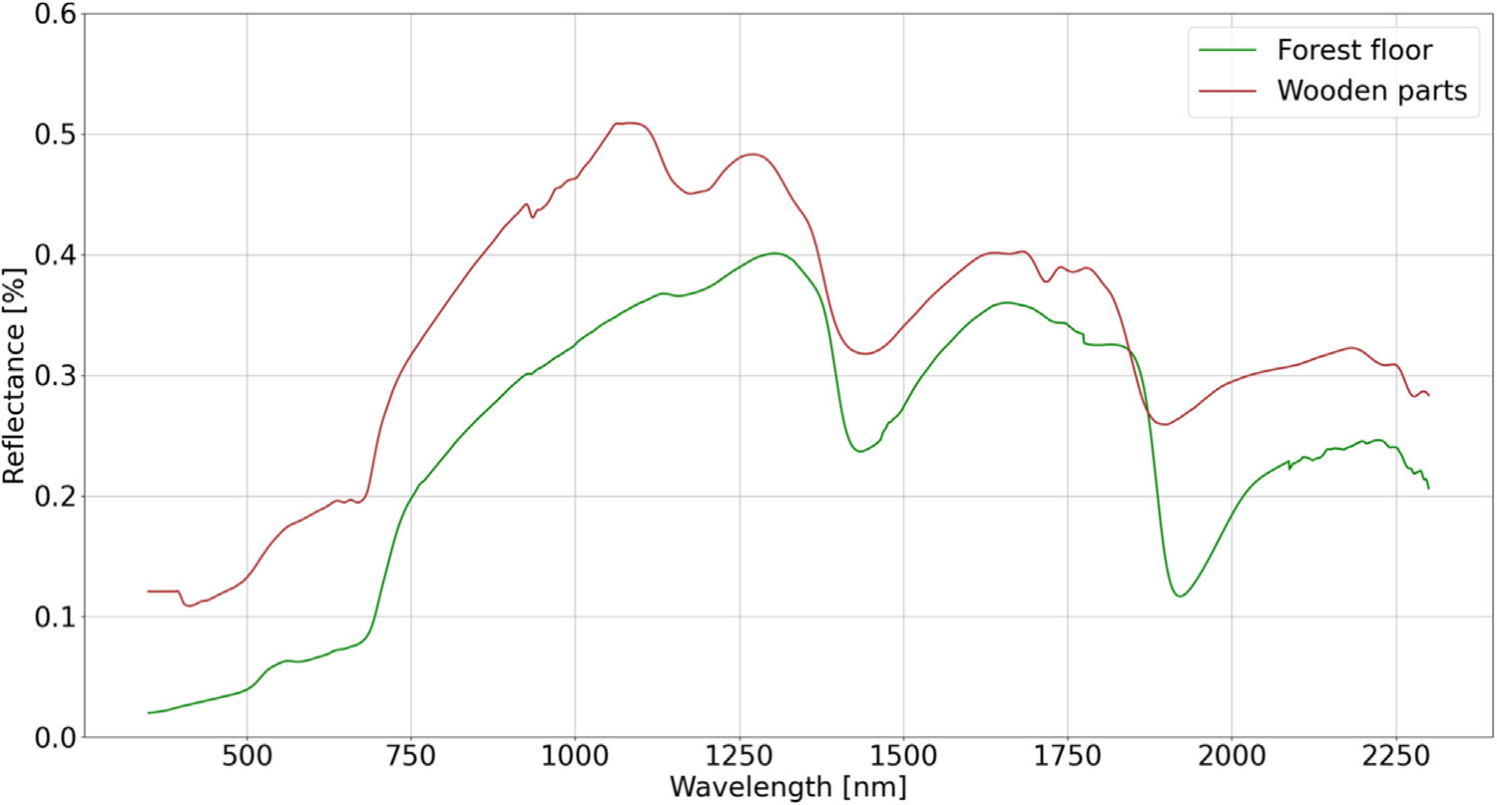
Spectra for forest floor and wooden parts used in simulations.

Optical properties for the forest floor and wooden parts (stem and branches) were measured at the Lanžhot site in the Czech Republic (48°40′46.974″ N, 16°57′19.174″ E, elevation 130–160 m a.s.l.) using a FieldSpec 4 spectroradiometer (Analytical Spectral Devices, Boulder, CO, USA) during the period from September 3 to September 12, 2019. The average spectra for forest floor and wooden parts were used to parameterize virtual scenes (Fig. 4). Optical properties for leaves were defined using PROSPECT-PRO model [13]. Combination configuration of the input parameters is described in Section 4.3.

The Bi-directional Light Mode (DART-Lux) [4] DART version 5.10.0 (build 1344), which employs the Monte Carlo method to trace light rays, was used for simulating canopy reflectance signature. The DART-Lux parameters settings utilized in the simulations are summarized in Table 4. To run the simulations with all the combinations of leaf traits, structural and scene parameters, we employed Sequence Launcher tool [12].

**Table 4:** Bi-directional Light (DART-Lux) Mode parameter settings used for simulation parameterization.

Parameter	Value
Maximal scattering order	80
Target pixel size [m]	0.2
Maximal rendering time per image [s]	720
Target sample density per pixels [images]	50
Number of repetitions of scene	3
Atmospheric radiation mode	Hybrid
Sampler	Sobol
Russian Roulette Acceleration	Yes

DART simulation outputs were limited to only Bi-directional Reflectance Factor (BRF) for the whole scene and images per individual wavelength with pixel size corresponding to a cell size to minimise computational demands. The canopy BRF outputs are in the spectral range from 450 nm to 2300 nm, with a 2-nm bandwidth (926 bands). In total, 3,456,000 unique simulations were generated. Due to the computational demands of the simulations (Table 5), we utilized the CESNET Metacentrum (https://www.metacentrum.cz/en/index.html).

**Table 5:** Computational demands of the LUT extraction steps to run a single sequence.

Step	CPUs	Avg. time (h)	RAM (Gb)	Disk space (Gb)
Simulation	20	43	50	500
Producing LUT from a single simulation	1	6.88	50	500

#### 4.3 Configuration of leaf traits combinations

**Fig. 5:**
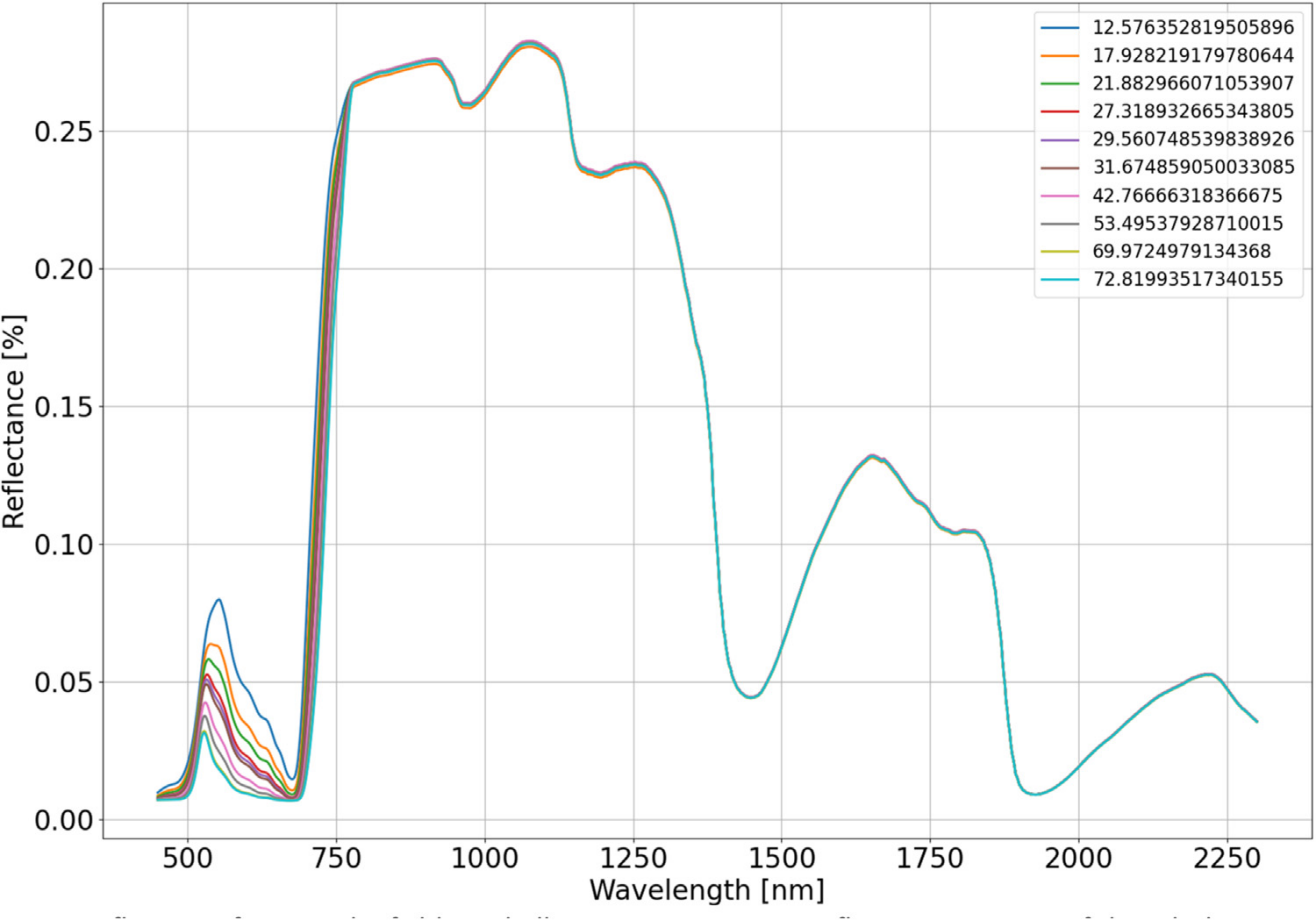
Influence of varying leaf chlorophyll content on canopy reflectance spectra of the whole scene, especially in the green absorption region and corresponding peak, with other leaf traits consistent. Each color represents a different leaf chlorophyll content value.

From the dataset of in-situ measured leaf traits we utilised a range of leaf traits, which consists of 26 trees including the following species: *Fagus sylvatica, Quercus robur, Quercus cerris L*., *Carpinus betulus, Fraxinus angustifolia, Tilia cordata,* which were repeatedly collected during three vegetation seasons (143 samples in total). From this dataset, we derived a random distribution for each leaf trait and generated 2000 combinations (Table 6). For illustration on how the range of a selected leaf trait influences the final canopy reflectance of the whole scene, the leaf chlorophyll content was selected (Fig. 5). Amount of leaf chlorophyll primarily affects the spectra around 550 nm, corresponding to the green absorption region, which is propagated from leaf to canopy spectra. As the chlorophyll content in leaves increases, both leaf and canopy reflectance decreases. Other regions of the spectra are not significantly influenced by the amount of leaf chlorophyll content.

**Table 6:** Random distribution characteristics of leaf traits for LUT.

Trait	Abbreviation	Distribution	Mean	Standard deviation	Combinations
Leaf chlorophyll content [µg/cm^2^]	Cab	normal	34.003	16.04424	2000
Leaf carotenoid content [µg/cm^2^]	Car	normal	5.427387	1.433608	
Leaf water content [cm]	Cw	normal	0.009018	0.00427	
Leaf dry matter content [g/cm^2^]	Cm	normal	0.0048	0.00134	
Structural parameter	N	normal	1.39	0.1757

#### 4.4 Post-processing of simulations to LUT

**Fig. 6:**
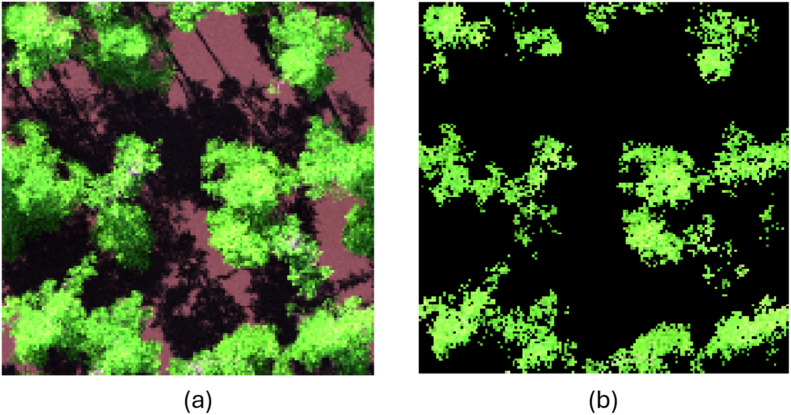
The RGB visualisation of one simulated Central European temperate broadleaf forests scene, (a) whole scene without masking, (b) only sunlit pixels, black pixels represent nodata.

The output from DART simulations consists of images in .mp# format for each spectral band, resulting in 926 images per simulation. These images were subsequently merged into a single hyperspectral cube per simulation using MATLAB (The MathWorks, Inc., Version R2020b) [14] code “merge_images.m” provided by the DART developers and adapted for this study. The hyperspectral cubes were then processed using a Python (Python Software Foundation, Version 3.6) [15] code “LUT_processing.py”, which performs the following steps: (i) links the hyperspectral cube with simulation trait parameters and submits trait values into “LUT_parameters.csv”, (ii) calculates the average value of all pixels (Fig. 6) in each band and submits the resulting values in “LUT_all_pixels.csv”, (iii) identifies and sets all non-sunlit and non-leaf pixels to a nodata value using local maximum in the Red Edge and thresholding, and (iv) calculates the average value of all sunlit leaf pixels (Fig. 6) in each band and submits the resulting values in “LUT_sunlit_pixels.csv”. Both files include all ∼3.5 million spectra (one row per spectrum). Each row represents reflectance spectra from 450 nm to 2300 nm with a 2-nm bandwidth (926 bands in total). Each row in the “LUT_parameters.csv” file provides a combination of parameters to the corresponding row in “LUT_all_pixels.csv” and “LUT_sunlit_pixels.csv”. Its header is provided on the first row and abbreviations are explained in Table 1 and in the file “LUT_variables_abbreviations.csv”. A subset of these files was created by randomly selecting 10,000 rows and saving them to separate files: “subset_LUT_parameters.csv”, “subset_LUT_all_pixels.csv” and “subset_LUT_sunlit_pixels.csv”. These files can provide better insight into the dataset. They can be valuable for users to set up and assess their code/program to use our dataset without the need to run the entire dataset each time.

## Limitations

• LUT were simulated only for European temperate broadleaf forests, therefore the application to other forest types could be inaccurate.
• Only healthy, mature trees were used for generating the LUT; young or old trees were not included in the scene setup.
• The minimum canopy cover considered is 60 %; therefore, sparse forests with a canopy cover below this threshold are not represented in the LUT.
• The trees included in the scene setup are from managed forests, no incline trees or trees from unmanaged plots were used.
• The forest floor was not simulated with any structural complexity but was instead represented as a homogeneous surface with predefined optical properties.

## Ethics Statement

The authors have read and follow the ethical requirements for publication in Data in Brief and confirm that the current work does not involve human subjects, animal experiments, or any data collected from social media platforms.

## Credit Author Statement

**Tomáš Hanousek**: Methodology, Data curation, Software, Visualization, Writing – original draft, Writing – review & editing. **Terézia Slanináková**: Software, Validation, Data curation, Writing – review & editing. **Tomáš Rebok**: Resources, Supervision, Writing – review & editing. **Růžena Janoutová**: Conceptualization, Methodology, Data curation, Supervision, Writing – review & editing.

## Data Availability

National RepositoryA dataset of hyperspectral Look-Up Tables for 3.5 million traits and structural combinations of Central European temperate broadleaf forests (Original data).
